# Novel Insight into Functions of Transcription Factor EB (TFEB) in Alzheimer’s Disease and Parkinson’s Disease

**DOI:** 10.14336/AD.2022.0927

**Published:** 2023-06-01

**Authors:** Jing Yang, Wei Zhang, Shugeng Zhang, Ashok Iyaswamy, Jichao Sun, Jigang Wang, Chuanbin Yang

**Affiliations:** ^1^Department of Geriatrics and Shenzhen Clinical Research Centre for Geriatrics, Shenzhen People's Hospital, The Second Clinical Medical College, Jinan University, The First Affiliated Hospital, Southern University of Science and Technology, Shenzhen, China.; ^2^Department of Hepatobiliary Surgery, Anhui Province Key Laboratory of Hepatopancreatobiliary Surgery, The First Affiliated Hospital of USTC, Division of Life Sciences and Medicine, University of Science and Technology of China, Hefei, China.; ^3^School of Chinese Medicine, Hong Kong Baptist University, Hong Kong SAR, China.; ^4^Artemisinin Research Center, Institute of Chinese Materia Medica, China Academy of Chinese Medical Sciences, Beijing, China.

**Keywords:** TFEB (transcription factor EB), lysosome, autophagy, neurodegenerative disease, Alzheimer’s disease, Parkinson’s disease

## Abstract

A key pathological feature of neurodegenerative diseases (NDs) such as Alzheimer’s disease (AD) and Parkinson’s disease (PD) is the accumulation of aggregated and misfolded protein aggregates with limited effective therapeutic agents. TFEB (transcription factor EB), a key regulator of lysosomal biogenesis and autophagy, plays a pivotal role in the degradation of protein aggregates and has thus been regarded as a promising therapeutic target for these NDs. Here, we systematically summarize the molecular mechanisms and function of TFEB regulation. We then discuss the roles of TFEB and autophagy-lysosome pathways in major neurodegenerative diseases including AD and PD. Finally, we illustrate small molecule TFEB activators with protective roles in NDs animal models, which show great potential for being further developed into novel anti-neurodegenerative agents. Overall, targeting TFEB for enhancing lysosomal biogenesis and autophagy may represent a promising opportunity for the discovery of disease-modifying therapeutics for neurodegenerative disorders though more in-depth basic and clinical studies are required in the future.

## 1. Introduction

In the 1990s, TFEB (transcription factor EB) was originally identified as a protein containing helix-loop-helix (HLH) and leucine-zipper region, which recognizes E box sequence at promoter regions of heavy-chain immunoglobulin [1]. Recently, TFEB was found to be a key regulator of lysosomal biogenesis and autophagy [2, 3]. In normal conditions, TFEB is mainly located in the cytoplasm and exists in an inactive form [4]. Upon translocation from the cytoplasm into the nucleus, TFEB binds to the motif of the coordinated lysosomal expression and regulation (CLEAR) element to upregulate many genes responsible for lysosomal biogenesis and autophagy [5]. The cytoplasm or the nuclear localization of TFEB is mainly regulated by its phosphorylation status at certain Ser residues. A variety of kinases or phosphatases have been reported to regulate the phosphorylation status of TFEB by various mechanisms, including ERK2 (extracellular signal-regulated kinase 2), MTORC1 (mechanistic target of rapamycin complex 1) [6], GSK3β (glycogen synthase kinase 3 β) [3], Akt (protein kinase B) [7, 8], PKC (protein kinase C) [9], PP2A (protein phosphatase 2A) [10], calcineurin and GCN5 (general control non-repressed protein 5) [11].

The lysosome is an organelle for degrading and recycling misfolded and dysfunctional proteins, and it fuses with autophagosomes as autolysosomes to degrade sequestered cargos [12]. Activation of TFEB-mediated lysosomal biogenesis to degrade protein aggregates is beneficial to NDs that are characterized by the accumulation of protein aggregates, including Alzheimer’s disease (AD) [3] and Parkinson’s disease (PD) [13]. Notably, impairment of lysosomal biogenesis and autophagy has been reported to be associated with the progression of these NDs [4]. Therefore, activation of TFEB or increasing TFEB expression is a potential therapeutic for these NDs. In recent years, numerous small-molecule TFEB activators have been identified and some of them show promising neuroprotective effects in multiple animal models of AD and PD.

The aim of this review is to summarize the current knowledge of TFEB-mediated lysosomal biogenesis and autophagy in NDs such as AD and PD, providing novel insight into understanding the pathogenesis of neurodegenerative diseases and the therapeutic potential of TFEB activators. Here, we provide an updated comprehensive understanding of the molecular mechanisms of TFEB activation and its roles in regulating lysosomal biogenesis and autophagy, then discuss its association with NDs with a particular focus on AD and PD, the two most common types of neurodegenerations. Finally, we illustrate several current small molecular TFEB activators and highlight therapeutics potential for targeting TFEB in NDs.

**Figure 1.**
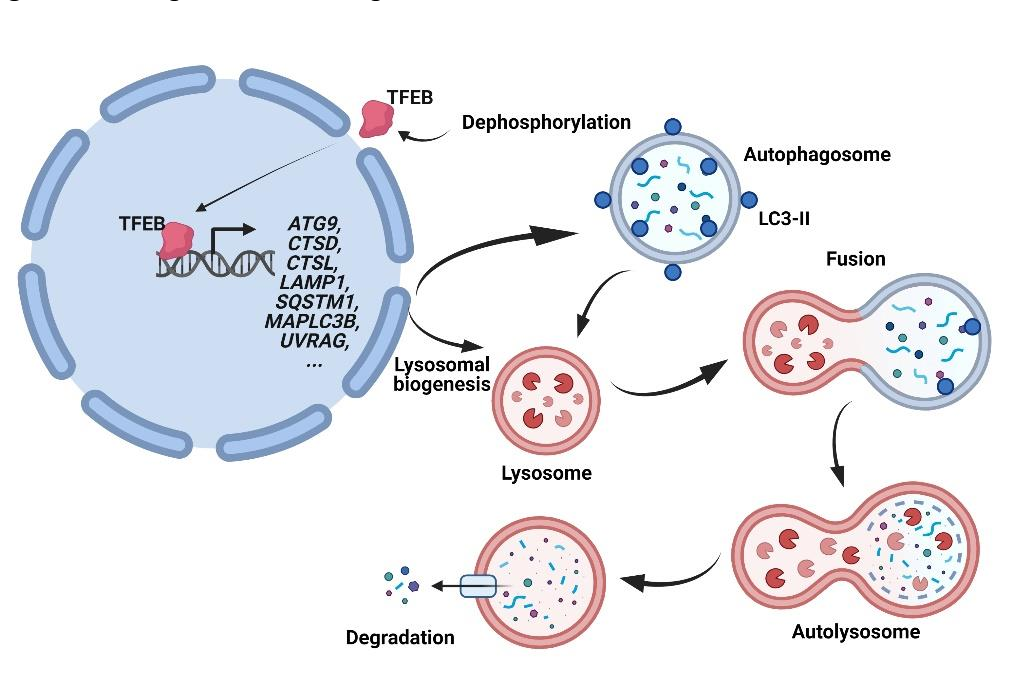
**TFEB promotes lysosomal biogenesis and autophagy.** TFEB is activated upon dephosphorylation, and it is then translocated into the nucleus to enhance lysosomal biogenesis and autophagy via upregulation of multiple genes in the autophagy-lysosomal pathway, including ATG9, CTSD, CTSL, LAMP1, SQSTM1, MAPLC3B, UVRAG, etc. Autophagy cargos sequestered in autophagosomes are degraded upon the fusion of autophagosomes with lysosomes to form autolysosomes.

## 2. Molecular mechanisms of TFEB regulation and its function

### 2.1. Transcriptional regulation of lysosomal biogenesis and autophagy by TFEB

Normally, TFEB is inactive and located in the cytoplasm [8]. Upon activation, TFEB translocates into the nucleus, and it then directly binds to the CLEAR sequence at promoter regions of multiple lysosomal and autophagy-associated genes, leading to upregulating the expression of these target genes and subsequent enhancement of lysosomal biogenesis and autophagy [5] (Fig. 1). Autophagy serves as a crucial catabolic process to degrade misfolded and toxic proteins via lysosomes [12, 14]. As a key transcriptional regulator of lysosomal biogenesis, TFEB promotes the expression of multiple genes involved in lysosomal biogenesis and autophagy, including *LAMP1*, *CTSD, CTSL*, *UVRAG*, *SQSTM1*, *MAPLC3B*, *ATG9* and others as shown in Figure 1 [2, 15]. As such, TFEB transcriptionally regulates autophagy by targeting multiple processes in autophagy, which include lysosomal biogenesis, autophagosome formation, and the fusion of autophagosomes with lysosomes. TFEB-mediated autophagy-lysosomal pathway (ALP) activation is different from canonical autophagy activators that only promote autophagosomes formation since a key role of TFEB activation is to increase the lysosomal functions. Since the impairment of lysosomal functions has been implicated in NDs, TFEB activators may show advantages for treating NDs compared with autophagy activators that promote the formation of autophagosomes.

**Figure 2.**
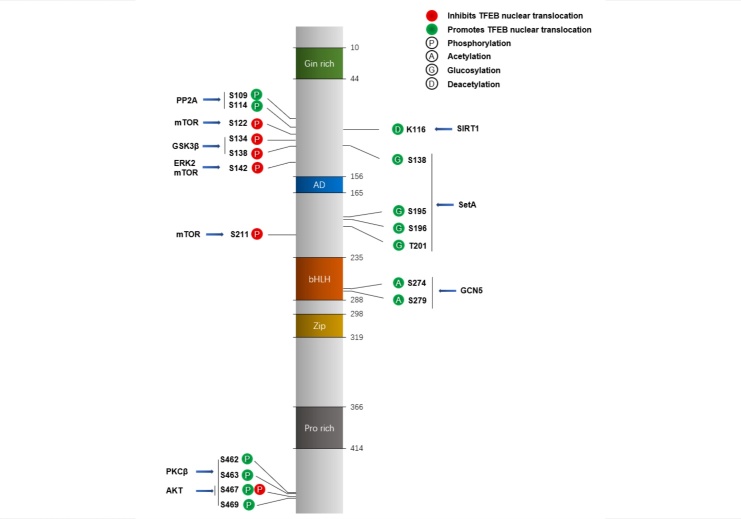
**Modification of TFEB by diverse kinases.** The subcellular localization or activity of TFEB is regulated by phosphorylation, acetylation, or glucosylation. Phosphorylation of TFEB by mTORC1 at Ser122, Ser142 and Ser211, by ERK2 at Ser142, or by GSK3β at Ser134 and S138, promotes its accumulation in the cytoplasm in an inactive form. In addition, AKT- inhibited TFEB nuclear translocation is via phosphorylating Ser467. In contrast, dephosphorylation of TFEB by PP2A at Ser109 and Ser114, and dephosphorylation of TFEB by calcineurin at Ser142 and Ser211 induce the nuclear accumulation of TFEB. In addition, TFEB activities can be regulated via deacetylation and glucosylation. For example, SIRT1 deacetylates TFEB at Lys116, resulting in the upregulation of TFEB transcriptional activity. GCN5 acetylates TFEB at Lys274 and Lys279, leading to decreased TFEB transcriptional activity, while SAHA (suberoylanilide hydroxamic acid) promotes TFEB activity via acetylation of TFEB at Lys91, Lys103, and Lys430. Apart from acetylation, the glucosyltransferase activity of SetA is required for the impairment of TFEB nuclear export by glucosylation at its Ser138 site.

### 2.2. Mechanisms for modulating TFEB activation

TFEB located either in the nucleus or the cytoplasm depends on its phosphorylation status. Normally, phosphorylated TFEB retains in the cytoplasm as an inactive form sequestered by the cytosolic chaperone 14-3-3 proteins [3, 5]. TFEB translocates from the cytoplasm into the nucleus to promote lysosomal biogenesis and autophagy upon dephosphorylation in response to multiple stimulus, which include lysosomal stresses [16], nutrient starvation [2], glucose deprivation [17] and cholesterol stress [18]. TFEB has several phosphorylation sites, which can be regulated by multiple kinases (e.g., mTORC1, ERK2, GSK3β) and phosphatases (e.g., calcineurin, PP2A) that are critical for its subcellular localization (Fig. 2). mTORC1, ERK2, and GSK3β phosphorylate and inactivate TFEB. In contrast, PP2A and calcineurin dephosphorylate and activate TFEB. Specifically, mTORC1 phosphorylates TFEB at Ser211 [19], Ser142 [16, 20] and Ser138 [20]. Interestingly, TFEB phosphorylation by mTORC1 is mediated by Rag GTPase and this is different from other canonical mTORC1 substrates such as S6K1 and 4E-BP1 [21]. ERK2, one of the extracellular signal-regulated kinases (ERKs), is another major candidate for the phosphorylation of Ser142 in TFEB [2]. GSK3β phosphorylates TFEB at Ser134 and Ser138 [3], PKCα and PKCδ induce TFEB translocation into the nucleus through inhibition of GSK3β-induced phosphorylation of TFEB [3]. In addition, Akt phosphorylates TFEB at Ser467 in an mTOR-independent manner to promote its cytoplasm accumulation [7]. Calcineurin binds to and dephosphorylates TFEB, leading to its nuclear accumulation [22]. Activation of PP2A was reported to dephosphorylate TFEB at Ser109, Ser114, Ser122 and Ser211, facilitating its nuclear translocation and activation [10]. Interestingly, glucosylation is another crucial regulator of TFEB. SetA, a *Legionella* effector, acts as a glucosyltransferase that modifies TFEB at multiple sites, including Ser138, Ser195, Ser196, Thr201, Ser203, and Thr208. In particular, its glucosyltransferase activity leads to the impairment of TFEB nuclear export by glucosylation at its Ser138 site, thereby promoting TFEB nuclear accumulation [23] (Fig. 2).

Apart from phosphorylation regulated subcellular localization of TFEB, other post-translational modifications such as acetylation also regulate TFEB’s activity. Inhibiting histone deacetylase by suberoylanilide hydroxamic acid (SAHA) results in the acetylation of TFEB at Lys91, Lys103 and Lys430, which subsequently enhances its transcriptional activity without affecting its nuclear accumulation [24]. Similarly, sirtuin 1 (SIRT1) deacetylates TFEB at Lys116, causes the increase of TFEB’s transcriptional activities in the nucleus [25]. In contrast, GCN5, a specific TFEB acetyltransferase, acetylates TFEB at Lys274 and Lys279, leading to the impairment of TFEB dimerization and subsequent disruption of the binding of TFEB to the promoters of its target genes [11]. These results suggest that post-translational modifications especially phosphorylation, acetylation and glucosylation are crucial for the regulation of TFEB nuclear accumulation or activities and the subsequent modulation of lysosomal biogenesis and autophagy (Fig. 2). Targeting these pathways may provide therapeutic potential for NDs that are associated with dysregulated ALP.

## 3. Lysosomal biogenesis and autophagy in neurodegenerative disease

### 3.1. Lysosomal functions and autophagy in AD and PD

A crucial hallmark of NDs is the accumulation of aggregated, and misfolded pathological proteins such as amyloid-β (Aβ) [26], and abnormally phosphorylated tau in AD [27, 28]; as well as α-synuclein in PD [29]. Autophagy plays a key role for the degradation of these toxic protein aggregates. Notably, dysfunction of the autophagy-lysosomal pathway is linked to the pathogenesis of various NDs [30] caused by genetic mutation and elevated aggregation of pathological proteins, which may further disrupt the autophagy-lysosomal pathway, and thus form a vicious cycle [31, 32]. Interestingly, clearance of pathological proteins alleviates disease pathologies by inducing lysosomal biogenesis and autophagy [33-35], thus enhancing autophagy shows great promise for treating NDs such as AD and PD.

### 3.2. AD

As the main kind of dementia and the most common neurodegenerative disorder, AD affects approximately 42.3 million people around the world. This number may increase to 81 million by 2040 [36]. Key features of AD are the accumulation of extracellular Aβ and intracellular hyperphosphorylated tau, which subsequently aggregates into amyloid plaques and neurofibrillary tangles, respectively, and eventually, induces synaptic toxicity and loss of neurons [37]. Increasing evidence has revealed that the dysfunction of the autophagy-lysosomal pathway in AD patients and AD animal models contributes to disease progression [38, 39].

Mutations in *PSEN1*, *PSEN2* and *APP* genes are responsible for early-onset familial AD. Lysosomal proteolysis is disrupted in early-onset AD with *PSEN1* mutation [40]. The amyloid precursor protein (APP) is cleaved by β-secretase to form β-C-terminal fragment (β-CTF) and soluble βAPP (β-sAPP), leading to the formation of Aβ_40_ and Aβ_42_ and the subsequent amyloid plaques [41]. Elevated β-CTF is involved in lysosomal pH elevation and aggregation of substrates, thus leading to dysfunction of lysosomes [42]. *APOE* e4 allele (*APOE4*) is a key risk gene for the onset of AD [43], and AD patients with homozygous *APOE4* exhibits lower levels of LAMP2, LC3-II, and SQSTM1(p62) in brains [44], indicating the disruption of the autophagy-lysosomal pathway. Several autophagy-lysosome-related genes are also involved in AD. For instance, the deletion of *Beclin 1* (*Becn1*), a key gene for autophagy, results in neurodegeneration with reduced autophagy [45]. Overexpression of *Becn1* ameliorates the amyloid pathology in AD mice via autophagy induction [45]. Moreover, a *Becn1* mutation F121A-mediated hyperactive induction of autophagy in AD mice decreases Aβ accumulation [46]. Similarly, reduced expression of a key autophagic gene *NRBF2* has been found in the hippocampus of AD mice and AD patient, and NRBF2 is associated with AD progression via autophagy regulation [47-49], providing another evidence for the impairment of autophagy in AD progression. Elevating the expression of other autophagic proteins including TFEB, LC3B-II and SQSTM1 also significantly reduced in AD animal models [50, 51].

Tau specifically binds to axons to assemble and stabilize microtubules [41]. Tau can be phosphorylated by GSK3β, CDK5, JNK, and α-Ⅰ-antichymotrypsin at several sites [52-55]. Hyperphosphorylated tau promotes the formation of neurofibrillary tangle in AD [56, 57]. It has been reported that phosphorylated tau is co-localized with accumulated autophagic vesicles in the brains of AD patients, indicating the association between tauopathy and dysfunction of autophagy [58]. Furthermore, phosphorylated Tau also compromises autophagy and mitophagy [32, 59] Tau can also be phosphorylated by increasing the mTOR activity. On the contrary, inhibition of mTOR to induce autophagy and subsequent tau degradation is effective in alleviating tauopathies in multiple AD animal models [60].

Overall, these findings suggest that dysregulation of multiple stages of the autophagy-lysosome pathway such as autophagosome function, and lysosomal dysfunction is involved in AD. Interestingly, a recent study highlighted that the failure of autolysosome acidification is responsible for AD pathogenesis such as plaque formation [61], highlighting the critical roles of lysosomal functions for AD therapeutics. However, a majority of studies have mainly focused on neuronal autophagy in AD, and accumulating evidence has revealed that glia cells are also crucial for AD pathogenesis. Therefore, future studies to dissect the roles of glia autophagy for AD progression may provide novel information for understanding the roles of autophagy in AD pathogenesis and progression.

### 3.3. PD

PD is the second most common neurodegeneration. According to a report in 2016, the number of global patients with PD has risen to 6.1 million, which was more than two times of the number in 1990 [62]. Unfortunately, currently drugs cannot stop or reverse disease progression and a variety of efforts have been made to identify novel potential therapeutics [63-66]. PD is characterized by the accumulation of α-synuclein, the main component of the Lewy body [67, 68]. Mutation in the gene coding α-synuclein, *SNCA*, is responsible for both familial and sporadic PD [29, 69]. Accumulating evidence has shown the critical role of autophagy in the clearance of aggregated α-synuclein in PD [34, 70]. As a result, degradation of α-synuclein by autophagy has become a potentially promising therapeutic target for PD.

The pathogenic α-synuclein mutants affect multiple stages of autophagy-lysosomal pathways. Firstly, A53T and A30P (two α-synuclein mutants) act as uptake blockers of the lysosomal membrane, resulting in impaired lysosomal functions [71]. Secondly, overexpression of A30P and A53T impair lysosomal functions by alkalinizing lysosomal PH value and disrupt endoplasmic reticulum (ER) - lysosomal Ca^2+^ signaling [72]. In addition, α-synuclein can disrupt the fusion of the autophagosomes with lysosomes by decreasing the levels of v-SNARE protein SNAP29, resulting in autophagic flux inhibition [73]. Lysosomes can also behave as the cargos for transferring α-synuclein among cells [74], and impairment of lysosomal functions may further aggravate the propagation of α-synuclein. Another example of the association between PD and lysosomes is that mutation in *ATP13A2*, a gene that encodes the lysosomal ATPase, leads to lysosomal dysfunction and PD progression [75].

Accumulated evidence has showed that upregulation of autophagy ameliorates PD-related pathologies [34, 70, 76]. Overexpression of *Becn1* to induce autophagy promotes α-synuclein degradation both *in vitro* and *in vivo*, which subsequently alleviates PD pathology in mice [77] [78]. TFEB, another crucial autophagic gene, is involved in the regulation of PD-related pathologies via autophagy. For instance, overexpression of *TFEB* in the midbrain ameliorates α-synuclein-induced toxicity via autophagy [13, 79] This amelioration may depend on oligodendroglial TFEB-targeted overexpression [80].

Overall, ALP plays a key role in promoting the degradation of α-synuclein, a key pathological protein in PD. Interestingly, neuroinflammation also plays key roles in PD pathogenesis and progression. Since ALP has also been implicated in regulating inflammation, apart from autophagy in neuron cells, future studies for dissection of the crosstalk between neuronal cells and glia cells, and how glia autophagy is regulated in PD will provide novel insight into PD pathogenesis.

## 4. TFEB signaling in neurodegenerative disease

As depicted above, impairment of lysosomal functions and autophagy has been linked to the progression of AD and PD [27, 81]. Here, as examples, we discuss the dysregulation of TFEB signaling in AD and PD and provide novel information of their pathogenesis and therapeutics.

### 4.1. TFEB signaling in AD

**Figure 3.**
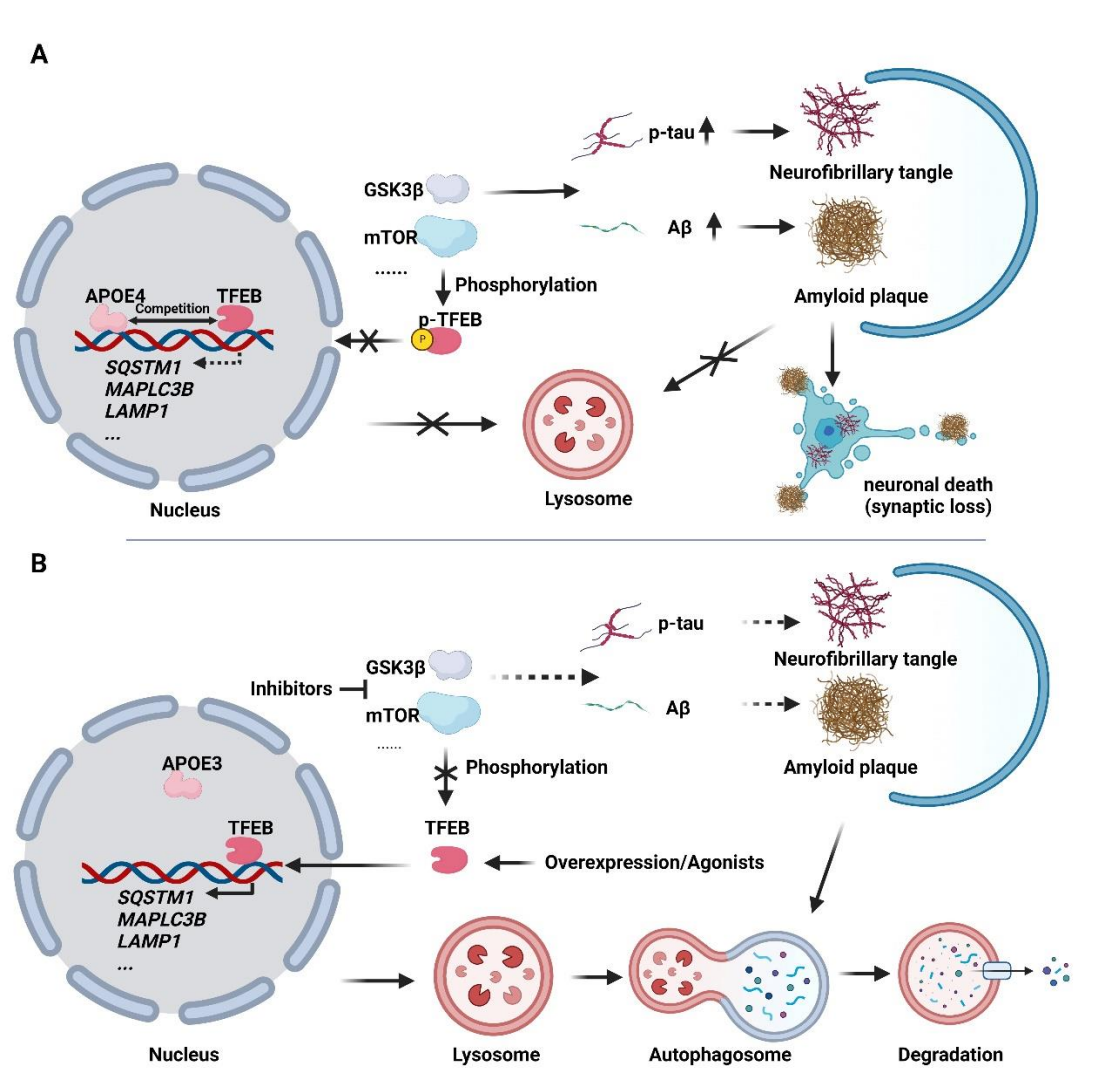
**TFEB-induced autophagy-lysosome pathway in AD.** (A) Dysregulation of TFEB-mediated signaling in AD. Normally, TFEB is phosphorylated by mTOR and GSK3β, leading to its inactivation in the cytoplasm. In addition, GSK3β is important for promoting tau phosphorylation. In the nucleus of AD models, APOE4 is mutated, and it competitively binds to CLEAR motif to disrupt TFEB-mediated lysosomal biogenesis and autophagy. As a result, the clearance of p-tau and Aβ is disrupted due to compromised TFEB functions. (B) Activation of TFEB enhances p-tau and Aβ clearance via lysosomal biogenesis and autophagy. Activated TFEB by mTOR and GSK3β inhibitor or overexpression of TFEB promotes lysosomal biogenesis and autophagy. APOE3, rather than APOE4, does not competitively bind to CLEAR motif, leading to the normal running of TFEB-mediated autophagy. As a result, p-tau and Aβ are degraded by TFEB-mediated autophagy.

As aforementioned, the key characteristics of AD are plaques formed by Aβ aggregates and neurofibrillary tangles, which are compromised hyperphosphorylated tau aggregation, accompanied by loss of synapses and neuron death [41, 82]. GSK3β is a key kinase for promoting tau phosphorylation [83] (Fig. 3A), and thus it is critical for AD pathogenesis. Interestingly, as aforementioned, TFEB has been identified to be phosphorylated by GSK3β at Ser134 and Ser138, resulting in cytoplasm sequestration of TFEB. Genetic or pharmacological inactivation of GSK3β promotes the nuclear accumulation of TFEB to induce lysosomal biogenesis and autophagy, leading to a reduction in Aβ_1-42_ and phosphorylated tau levels and subsequent amelioration of cognitive deficits in AD murine models [3, 84, 85] (Fig. 3B). Inhibition of mTOR signaling to activate TFEB-mediated lysosomal biogenesis and autophagy, leads to clearance of Aβ and tau, and improvement of cognitive function in AD animal models [86-88]. Apart from inhibition of negative regulators of TFEB, direct overexpression, and activation of TFEB also ameliorates AD-related pathologies [25, 89, 90] (Fig. 3B).

*APOE4* (apolipoprotein E4) is a major risk gene for AD [36, 91]. People with homozygous *APOE4* mutation have a risk of more than 50% for the onset of AD [91]. APOE4 mutation markedly exacerbates AD-related pathologies, including increased Aβ secretion and tau levels [92, 93]. It has been reported that APOE3, a protective isoform of APOE against AD, has a weak ability to bind to the CLEAR motif (Fig. 3B). In contrast, APOE4 competitively binds to CLEAR motif and suppresses the binding of TFEB, resulting in decreased transcriptional expression of multiple TFEB target genes such as LC3, SQSTM1, and LAMP2 [94] (Fig. 3A).These results highlight the critical roles of a key AD risk gene *APOE4* in the modification of TFEB activities. TFEB signaling in AD is summarized in Figure 3. Overall, these results highlighted the critical role of TFEB in AD.

### 4.2. TFEB signaling in PD

The cytoplasm accumulation of TFEB and subsequent impairment of lysosomal functions and autophagy have been identified in PD mouse models and PD patients [65, 81, 95]. To be specific, a PD hallmark protein α-synuclein sequesters TFEB in the cytoplasm and thus inhibits TFEB-mediated lysosomal biogenesis and autophagy [96]. Overexpression of TFEB rescues neuronal function in PD animal models [13]. In addition, overexpression of TFEB in oligodendrocytes induces lysosomal biogenesis and autophagy, and thus ameliorates aggregates of α-synuclein in PD rats [80].

**Figure 4.**
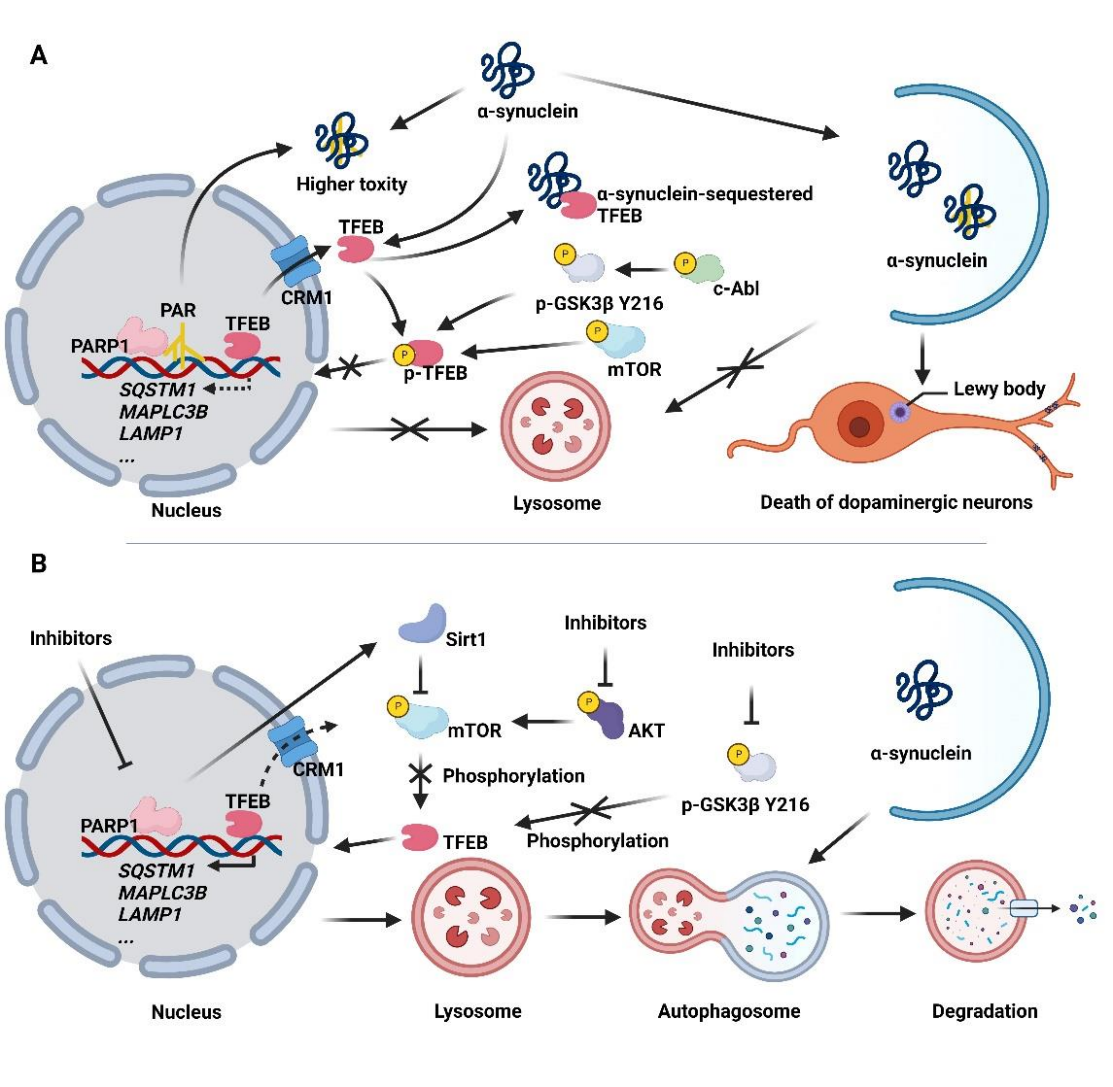
**TFEB-mediated autophagy-lysosomal pathway in PD.** (A) Dysfunction of TFEB-mediated lysosomal autophagy in PD. α-synuclein- sequestered TFEB in the cytoplasm. PARP1 induces the formation of PAR, leading to the formation of higher toxicity of α-synuclein. Activation of mTOR and GSK3β via phosphorylation at Y216 by elevated c-Abl in PD induces TFEB phosphorylation and thus impairs TFEB-mediated lysosome biogenesis and autophagy. These results lead to impairment of the clearance of α-synuclein via TFEB-mediated lysosomal autophagy. (B) Activation of TFEB-mediated lysosomal autophagy in PD. PARP1 inhibition induces Sirt1-mediated mTOR inactivation and subsequently TFEB dephosphorylation and activation, PARP1 inhibition also compromises TFEB nuclear export via disrupting the interaction of TFEB and CRM1. In addition, inhibition of GSK3β also promotes TFEB dephosphorylation and activation. These results lead to the induction of TFEB-mediated lysosomal autophagy, and thus accelerate α-synuclein degradation.

As aforementioned, GSK3β phosphorylates TFEB at several sites and inhibits the nucleus accumulation of TFEB. It was reported that inhibition of the activity of GSK3β ameliorates PD-associated pathologies partially via activating TFEB-mediated lysosomal biogenesis and autophagy. For example, nonreceptor tyrosine kinase Abelson (c-Abl) is increased in PD patients and PD animal models, pharmacological inhibition of c-Abl inhibits GSK3β activity, and thus activates TFEB-mediated lysosomal biogenesis to promote α-synuclein degradation and alleviates PD pathology [97]. Apart from GSK3β, inactivation of AKT-mTOR pathways also results in TFEB nuclear translocation and subsequent enhancement of lysosomal biogenesis and autophagy, which ultimately induces the clearance of α-synuclein and ameliorates MPP^+^-induced cell death [98].

In addition, poly (ADP-ribose) polymerase 1 (PARP1), an enzyme associated with DNA damage, was reported to be activated in PD animal models. PARP1 induces the formation of poly (ADP-ribose) polymers (PAR) and exacerbates the toxicity of α-synuclein [99]. Inhibition of PARP1 improves TFEB nuclear translocation and accumulation via SIRT1-mediated mTOR inhibition and reduces the interaction of TFEB with CRM1 (transporter chromosome region maintenance 1) to inhibit TFEB nuclear export, leading to TFEB-dependent autophagy [81]. TFEB signaling in PD is summarized in Figure 4. Overall, these studies underline the importance of TFEB signaling in PD.

**Figure 5.**
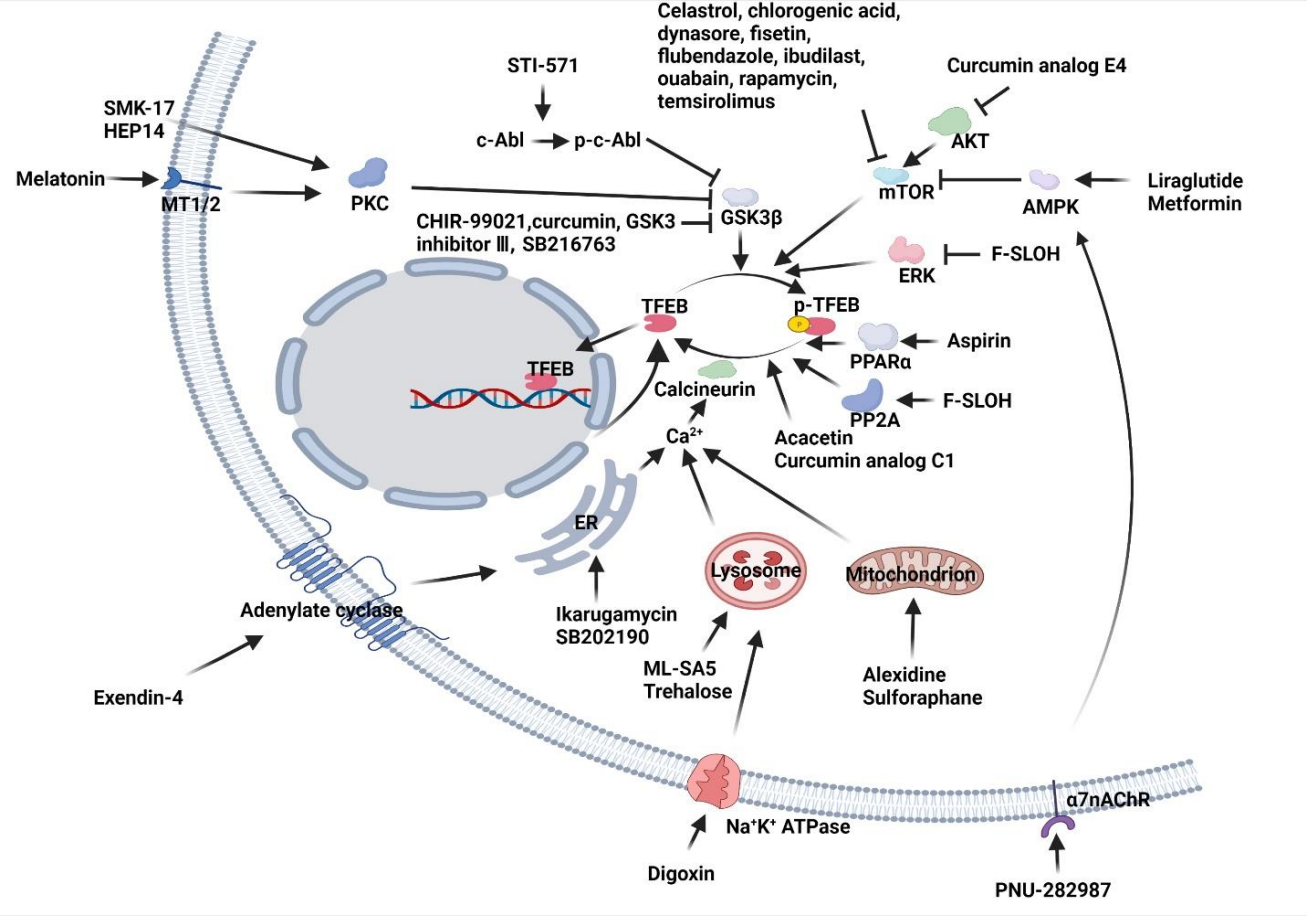
**Known small molecules that promote TFEB nuclear translocation.** TFEB can be activated through multiple pathways that are activated via different drugs. For example, melatonin, HEP14, SMK-17, STI-571, CHIR-99021, curcumin, GSK3 inhibitor III, and SB216763 induce TFEB nuclear translocation through the GSK3β-mediated pathway. Celastrol, chlorogenic acid, dynasore, fisetin, flubendazole, ibudilast, ouabain, rapamycin, temsirolimus, curcumin analog E4, PNU-282987, liraglutide, and metformin triggers TFEB nuclear translocation via mTOR signaling pathway. Exendin-4, ikarugamycin, SB202190, ML-SA5, trehalose, digoxin, sulforaphane, and alexidine induce calcium release and subsequently activate TFEB by calcineurin. In addition, aspirin induces TFEB nuclear translocation via PPARα activation. F-SLOH inhibits MAPK and activates PP2A, leading to TFEB dephosphorylation. Apart from those drugs, acacetin and curcumin C1 trigger TFEB translocation to the nucleus by mTORC1-independent pathway.

### 4.3. Therapeutic potential of targeting TFEB in neurodegenerative disease

TFEB nuclear translocation is critical for autophagy and lysosomal biogenesis. Hence, discovering drugs that induce TFEB nuclear translocation hold promising for potential therapies for NDs. As aforementioned, TFEB is inactivated via phosphorylation (Fig. 2), and thus, small molecules that are capable of promoting TFEB dephosphorylation all have the potential to induce the nuclear accumulation of TFEB and promote lysosomal biogenesis and autophagy. Such small molecule TFEB activators identified are summarized in Table 1 and Figure 5.

**Table 1:** Common drugs inducing TFEB nuclear translocation.

Name of drugs	Potential targets/ pathways involved in mediating TFEB activation	Appropriate concentration	Cell lines or animal models studied	Description
**Acacetin [112]**	Independent mTOR inhibition	50 μM	HeLa cells	Acacetin activated TFEB-mediated xenophagy against *Salmonella*
**Alexidine [118]**	PTPMT1	3.3 μM	HeLa cells	Alexidine activated TFEB nuclear translocation via mitochondrial Ca^2+^-mediated pathway.
**Aspirin [123]**	Transcriptional upregulation of TFEB via PPARα	10 μM	Primary cortical neurons	Aspirin activated PPARα and induces TFEB nuclear translocation.
**Celastrol [87]**	mTOR inhibition	>0.5 μM	HeLa cells	Celastrol induced TFEB nuclear translocation via mTOR inhibition.
**Curcumin analog C1 [89]**	Directly binds with TFEB	1.2 µM	N2a cells	Curcumin analog C1 activated TFEB to ameliorate AD-related pathologies independent of mTOR inhibition
**Digoxin [118]**	Na+, K+ ATPase Digoxin	370 nM	HeLa cells	Digoxin induced TFEB nuclear translocation via lysosomal Ca^2+^-mediated pathway.
**Dynasore [105]**	mTOR inhibition	50 µM	HEK 293 cells	Dynasore inhibited mTOR activity and activated TFEB-mediated ALP.
**Exendin-4 [121]**	RAPGEF4/EPAC2-calcium-calcineurin	25 nmol/kg	C57BL/6 mice	Exendin-4 activated TFEB nuclear translocation via RAPGEF4/EPAC2-mediated Ca^2+^ pathway.
**Fisetin [106]**	mTOR inhibition	>5 μM	T4 cells	Fisetin inhibited activity of mTOR, leading to the translocation of TFEB into nucleus.
**Flubendazole [107]**	mTOR inhibition	~16 μM	HeLa cells	Flubendazole inhibited the activity of mTOR by disrupting dynamic microtubules, and thus induced TFEB-mediated autophagy.
**F-SLOH [124]**	ERK inhibition	>12.5 μM	Microglia cells	F-SLOH inhibited MAPK and activated PP2A, leading to TFEB nuclear translocation and subsequent ALP.
**Genistein [125]**	/	150 μM	Huh7 cells HeLa cells SH5Y cells	Genistein-induced TFEB nuclear translocation and subsequent autophagy.
**GSK3 inhibitors VIII [85]**	GSK3 inhibition	5 μM	N2a cells	Calbiochem GSK3 inhibitors VIII induced TFEB nuclear translocation via inhibiting GSK3
**Gypenoside XVII [126]**	/	10 μM	PC12 cells	Gypenoside XVII initiated TFEB-mediated lysosomal biogenesis and subsequent lysosomal-autophagy.
**HEP14 [3]**	PKC-mediated GSK3β inhibition	20 μM	HeLa cells	HEP14 activated TFEB by PKC-mediated two different signalling cascades. In one way, PKC inactivated GSK3β and subsequently induced TFEB translocation into the nucleus.
**Ibudilast [108]**	mTOR inhibition	2 μM	HEK293 cells	Ibudilast induced TFEB nuclear translocation via inhibition of mTOR.
**Ikarugamycin [118]**	ER Ca2+ ATPase-mediated calcineurin	3.3 μM	HeLa cells	Ikarugamycin activated TFEB nuclear translocation via the ER-Ca^2+^ pathway.
**Kaempferide [127]**	mitochondrial ROS-mediated lysosomal Ca^2+^ efflux	20 μM	HEK293 cells	Kaempferide directly bound TUFM and induces TFEB nuclear translocation to alleviate metabolic dysregulation.
**Liraglutide [113]**	AMPK-MCOLN1-calcineurin	200 μg/kg	C57BL/6 mice	Liraglutide induced TFEB nuclear translocation via AMPK-mTOR pathway.
**Metformin [114]**	AMPK	125 μg/kg	C57BL/6 mice	Metformin activated AMPK and subsequently inactivated mTOR, inducing TFEB nuclear translocation.
**ML-SA5 [117]**	Activation of TRPML1	2 mg/kg	C57BL/6 mice	Activated TFEB nuclear translocation via TRPML1-Ca2^+^ pathway.
**Ouabain [109]**	mTOR inhibition	0.5 μM	Mouse primary cortical neurons	Ouabain induced TFEB nuclear translocation via inhibition of mTOR.
**Rapamycin [111]**	mTOR TRPML1	5 µM	HeLa cells	Rapamycin activated TFEB by TRPML1-Ca2^+^ and mTOR pathways.
**SB202190 [122]**	Calcium-mediated calcineurin activation	>5 μM 20 µM	HeLa cells HEK293 cells	SB202190 activated TFEB-mediated ALP independent of p38 inhibition
**SB216763 [100]**	GSK3β inhibition	10 μM	Neural stem cells	SB216763 inactivated GSK3β to activate TFEB and subsequent autophagy. This inhibition was associated with unfolded protein response, glycogen and differentiation of neural stem cells.
**SMK-17 [104]**	PKC activation	10 µM	PC12D cells	SMK-17 induced TFEB nuclear translocation dependent on PKC.
**STI-571 [97]**	c-Abl-mediated GSK3β inhibition	5 µM	SN4741 cells	STI-571 inactivated c-Abl, and consequently inhibited the activity of GSK3β, leading to TFEB nuclear translocation.
**Sulforaphane [120]**	Mitochondria (ROS)-calcium	>10 μM	HeLa cells	Sulforaphane activated TFEB nuclear translocation via ROS upregulation
**Temsirolimus [111]**	mTOR inhibition TRPML1	5 µM	HeLa cells	Temsirolimus activated TFEB by TRPML1-Ca2^+^ and mTOR pathways.
**Trehalose [119, 128]**	calcium-calcineurin activation	100 mM	NSC34 cells AML12 cells	Trehalose induced TFEB nuclear translocation by its dephosphorylation.

Recently, multiple small molecules that activate TFEB have been identified. For example, curcumin, CHIR-99021, GSK3β inhibitor VIII and SB216763 induce TFEB nuclear translocation via inhibiting GSK3β [85, 97, 100, 101]. Activation of PKC by melatonin, SMK-17 and HEP14 also leads to TFEB activation via GSK3β inhibition [3, 102-104]. In addition, inhibition of mTOR by celastrol, chlorogenic acid, dynasore, fisetin, flubendazole, ibudilast, ouabain, rapamycin, and Curcumin analog E4, and temsirolimus also activates TFEB [87, 98, 105-111]. Beyond that, acacetin, curcumin analog C1 can also induce TFEB nuclear translocation via TFEB dephosphorylation [89, 112]. Unlike other curcumins, curcumin analog C1 activates TFEB by directly binding to TFEB, which is independent on mTORC1 inhibition [89]. Acacetin was reported to activate TFEB independent of the mTOR signaling pathway [112]. Furthermore, AMPK activation by PNU-282987, liraglutide and metformin induces mTOR inhibition and enhances TFEB-mediated lysosomal biogenesis and autophagy [113-116].

As one of the upstream pathways of TFEB, Ca^2+^-dependent calcineurin activation leads to TFEB dephosphorylation and activation. A variety of small molecules activate TFEB via a calcium-dependent calcineurin pathway. For instance, ML-SA5, a mucolipin 1 (ML1) agonist, induces ML1-mediated Ca^2+^ release from lysosomes [117]. In addition, digoxin and trehalose induces TFEB nuclear translocation via a lysosomal Ca^2+^-mediated pathway [118, 119]. Alexidine, a protein tyrosine phosphatase mitochondrial 1 (PTPMT1) inhibitor, increases the cytoplasm Ca^2+^ levels [118], sulforaphane induces Ca^2+^ release from mitochondrial, leading to TFEB nuclear translocation [120]. The endoplasmic reticulum (ER) is a major place for Ca^2+^ storage. Activation of ER-mediated Ca^2+^ release by exendin-4, ikarugamycin and SB202190 was reported to induce TFEB dephosphorylation and activation [118, 121, 122]. Overall, multiple small molecule TFEB activators have been identified, which modulate the activity of TFEB via regulating various signal pathways that mediate post-modifications of TFEB.

Interestingly, some small molecule TFEB activators are effective in promoting the degradation of toxic proteins and alleviating the pathologies in multiple NDs, including AD, and PD [89, 129]. Multiple TFEB activators have been reported to ameliorate AD-related pathologies function via mTORC1 inhibition, including celastrol [87], chlorogenic acid [88], fisetin [106], flubendazole [107], ouabain [109] and pseudo-ginsenoside-F11 [130]. In addition, it has been demonstrated that inhibition of mTOR by small molecules is also beneficial to other NDs, such as ibudilast in ALS and dynasore in HD [105, 108]. Metformin is a well-studied drug for the treatment of AD in animal models [131]. In recent years, metformin has been reported to induce TFEB nuclear translocation via AMPK-mTOR signaling pathway [132]. Apart from mTOR, GSK3β is also a well-known target for drugs in regulating TFEB-mediated lysosomal autophagy in NDs. Activation of PKC can inactivate GSK3β, leading to translocation of TFEB into the nucleus [3]. Similar induction also occurs, which is mediated by inhibitors of other negative regulators of TFEB (for example, SB216763, a GSK3β inhibitor, and MK2206, an Akt inhibitor). Some of these inhibitors further ameliorate the pathologies of AD [60, 107] and PD [34, 97]. Apart from these targets, a great number of papers have reported that peroxisome proliferator-activated receptors (PPARs) is associated with TFEB-mediated lysosomal functions and autophagy [51, 123, 133]. Activation of peroxisome proliferators-activated receptors α (PPARα) by gemfibrozil and Wy14643 was demonstrated to enhance TFEB-mediated autophagy, resulting in reduced AD-associated pathologies and cognitive deficits [51]. Similarly, aspirin and cinnamic acid activate PPARα and induce TFEB-mediated lysosomal biogenesis, leading to the amelioration of pathologies of AD [123, 134]. PPARγ co-activator 1α (PGC-1α) can reduce HD-related proteotoxicity by activating TFEB [135]. In addition, small molecule compounds, including curcumin analog C1 [89] and trehalose [136], are protective against AD through TFEB activation. Many drugs reported for targeting TFEB to mitigate NDs are summarized in Table 2. Notably, neuroinflammation is also associated with the progression of NDs such as AD and PD. For instance, inhibition of inflammation especially NF-κB-mediated neuroinflammation by many compounds such as Mucuna pruriens [64, 137], ursolic acid [138, 139] and chlorogenic acid [140] shows promising [141] anti-PD effects. Since TFEB has also been implicated in regulating inflammation[142], future studies to dissect the roles of TFEB-associated inflammation in NDs may provide insight for our understanding of disease pathogenesis. Overall, activating TFEB represents a promising strategy for treating NDs.

**Table 2:** Common drugs or small molecules inducing TFEB-mediated autophagy and alleviating disease pathology in NDs.

Name	Mechanism of action for TFEB activation	Target disease(s) and references	Efficacy in Disease Models
**Aspirin**	Transcriptional upregulation of *Tfeb* via PPARα	AD [123]	Alleviated AD associated pathology in 5XFAD mice
**Celastrol**	mTORC1 inhibition	AD [87, 143]	Ameliorated Tau pathology in P301S Tau and 3XTg mice.
**CHIR-99021**	GSK3β inhibition	PD [97]	Reversed MPP^+^-induced autophagy-lysosomal dysfunction
**Chlorogenic acid**	mTOR inhibition	AD [88]	Alleviated cognitive deficiency in APP/PS1 mice
**Cinnamic acid**	Transcriptional upregulation of *Tfeb* via PPARα	AD [134]	Decreased amyloid plaques in 5XFAD mice
**Curcumin**	GSK3β inhibition	AD [101]	Ameliorated amyloidogenesis and antioxidants in neuronal cells
**Curcumin analog C1**	Activation of TFEB independent of mTORC1 inhibition	AD [89], PD [129]	Ameliorate AD-related pathologies in 5XFAD, 3XTg and P301S tau mice, and alleviated cell death in 6-OHDA-induced PD animal models.
**Curcumin analog E4**	mTORC1 inhibition	PD [98]	Alleviated neuronal cell death in PD cell models
**Fisetin**	mTORC1 inhibition	AD [144]	Promoted pathological Tau degradation
**F-SLOH**	ERK1/2 inhibition	AD [124]	Alleviated AD-related pathologies in 5XFAD and 3XTg mice.
**Gemfibrozil**	Transcriptional upregulation of *Tfeb* via PPARα	AD [51]	Improvement of AD-associated pathologies in APP/PS1 mice.
**HEP14**	PKC-mediated GSK3β inhibition	AD [3]	Reduce Aβ plaque in APP/PS1 mice
**Ouabain**	mTORC1 inhibition	AD [109]	Alleviated tau pathologies in fly and mice.
**Pseudoginsenoside-F11**	mTORC1 inhibition	AD [130]	Alleviated oligomeric Aβ-induced protein endosome-lysosomal dysfunction in microglia cells
**Qingyangshen**	Transcriptional upregulation of Tfeb via PPARα	AD [145]	Ameliorated AD-related pathologies in 3XTg mice
**STI-571**	GSK3β inhibition	PD [97]	Reversed MPP^+^-induced ALP dysfunction and cell death in neurons
**Veliparib**	SIRT1 mediated mTORC1 inhibition	PD [81]	Promoted α-synuclein degradation and alleviated neurodegeneration in PD mice
**Wy14643**	Transcriptional upregulation of *Tfeb* via PPARα	AD [51]	Alleviated AD-associated pathologies in APP/PS1 mice
**Trehalose**	calcineurin activation	AD [146, 147], PD [136, 148, 149]	Trehalose activated TFEB-dependent autophagy to degrade misfolded proteins and alleviated disease pathologies.

## 5. TFEB and aging

Aging is a process of physiologically chronic functional decline, and it is a leading factor in the pathology of many NDs [150], including AD [151] and PD [152]. Impairment of lysosomal functions occurs during aging, resulting in cholesterol crystallization, inflammasome activation [153, 154] and inhibition of quiescent neural stem cell activation [155]. As a master transcriptional regulator of lysosomes, TFEB plays a crucial role in aging and NDs. Its homolog, BHLH domain-containing protein (HLH-30) in *Caenorhabditis elegans* (*C. elegans*), can also regulate lysosomal function and autophagy [156, 157]. Protein homeostasis is disrupted during aging, and nuclear translocation of HLH-30/TFEB can activate a compensatory regulation in response to the aging-associated disruption of protein homeostasis [158]. HLH-30/TFEB has been reported to extend the longevity of *C. elegans* and alleviate metabolic diseases by upregulating autophagic genes [156]. Germline deficiency prolonged the lifespan of *C. elegans* [159], and HLH-30/TFEB regulated this process by upregulating Mondo/Max-like complex1 [160]. In addition, dietary restriction, a widely-used method for extending lifespan, has also been shown to promote TFEB nuclear translocation in mice [156]. Three TFEB agonists, including digoxin, ikarugamycin and alexidine dihydrochloride, have been demonstrated to ameliorate oleic acid-induced lipid accumulation in mice, and prolong the lifespan of *C. elegans* [118]. As a result, HLH-3/TFEB is critical for lifespan extension. Further studies on the roles of TFEB in extending lifespan in mammals are strongly required. Additionally, aging is a key factor for neurodegenerative disease, future studies dissecting the molecular connections between TFEB, aging, and neurodegenerative disease may provide novel insight into the discovery of agents for aging and NDs.

## 6. Conclusion and perspective

The hallmarks of NDs including AD and PD are aggregated and misfolded proteins, which compromise normal TFEB functions and its involved lysosomal functions and autophagy. Additionally, several AD and PD-associated risk genes also affect TFEB-mediated ALP. Dysregulated lysosomal functions and autophagy have been reported to be closely relevant to the onset and progression of many NDs such as AD and PD [161]. Thus, inducing the degradation of these protein aggregates such as Aβ plaques in AD and α-synuclein in PD is one of the promising therapies. As described above, enhancement of the activity of TFEB induces lysosomal biogenesis and subsequent autophagy, leading to the degradation of the above protein aggregates and alleviation of the pathologies in multiple PD/AD animal models. Interestingly, many small molecules have been identified to show promising effects in activating TFEB and promoting lysosome-mediated degradation of protein aggregates, which consequently alleviate disease pathology in multiple animal models of AD and PD [3, 51, 129]. Hence, TFEB has been regarded as a potential promising target for the treatment of AD and PD. Discovering and developing novel small molecule TFEB activators that promote the degradation of toxic proteins and alleviate the pathologies in NDs will advance the development of potential therapeutics for NDs.

However, several questions are still to be clarified in the future. For instance, the specificity of TFEB activators is remained to be elucidated as most of the small molecules have off-targets effects. Targeting Nrf2 signaling also show promising effects in treating NDs including AD and PD. Since recent study showed that TFEB activated Nrf2 [162] and the canonical Nrf2 activator sulforaphane also increased TFEB-mediated lysosomal biogenesis, whether neuroprotective effects of multiple TFEB activators in NDs are solely dependent on TFEB activation needs further in-depth investigation. Additionally, potential side effects of long-term autophagy activation should be examined in the future since overactivation of autophagy may be detrimental to neurons. Currently, the majority of TFEB activators inhibit mTOR and the discovery of mTOR-independent TFEB activators may be preferred because mTOR plays a major role in regulating normal neuron function. Furthermore, the crosstalk between TFEB-mediated autophagy and other selective autophagy such as mitophagy, chaperone-mediated autophagy (CMA), ER-mediated autophagy, or pexophagy is not yet completely elucidated. Whether TFEB exerts protective effect in NDs through those forms of autophagy needs further investigation. For instance, mitophagy is an important process for the degradation of injured mitochondria. It has been reported that activation of TFEB by inhibiting mTOR can also induce mitophagy [163, 164]. PGC-1α is a critical regulator of mitophagy and PGC-1α also activated TFEB-mediated mitophagy [165], whether TFEB plays a role in mitophagy and its role in neurodegenerative disease are yet to be determined. Nevertheless, targeting TFEB-mediated lysosomal biogenesis and autophagy is a promising therapeutic for current incurable NDs.
